# Vascular therapy for Duchenne muscular dystrophy (DMD)

**DOI:** 10.12703/r/12-3

**Published:** 2023-02-21

**Authors:** Sangharsha Thapa, Shaymaa Elhadidy, Atsushi Asakura

**Affiliations:** 1 Stem Cell Institute, Paul & Sheila Wellstone Muscular Dystrophy Center, Department of Neurology, University of Minnesota Medical School, MN, USA

**Keywords:** Muscular dystrophy, endothelial cell, VEGF, VEGFR, satellite cell, muscle regeneration

## Abstract

Duchenne muscular dystrophy (DMD) is a progressive disease characterized by the wasting of the muscles that eventually lead to difficulty moving and, ultimately, premature death from heart and respiratory complications. DMD deficiency is caused by mutations in the gene encoding *dystrophin*, which prevents skeletal muscle, cardiac muscle, and other cells from producing the functional protein. Located on the cytoplasmic face of the plasma membrane of muscle fibers, dystrophin serves as a component of the dystrophin glycoprotein complex (DGC), mechanically reinforces the sarcolemma, and stabilizes the DGC, preventing it from contraction-mediated muscle degradation. In DMD muscle, *dystrophin* deficiency leads to progressive fibrosis, myofiber damage, chronic inflammation, and dysfunction of the mitochondria and muscle stem cells. Currently, DMD is incurable, and treatment involves the administration of glucocorticoids in order to delay disease progression. In the presence of developmental delay, proximal weakness, and elevated serum creatine kinase levels, a definitive diagnosis can usually be made after an extensive review of the patient’s history and physical examination, as well as confirmation through muscle biopsy or genetic testing. Current standards of care include the use of corticosteroids to prolong ambulation and delay the onset of secondary complications, including respiratory muscle and cardiac functions. However, different studies have been carried out to show the relationship between vascular density and impaired angiogenesis in the pathogenesis of DMD. Several recent studies on DMD management are vascular targeted and focused on ischemia as a culprit for the pathogenesis of DMD. This review critically discusses approaches—such as modulation of nitric oxide (NO) or vascular endothelial growth factor (VEGF)-related pathways—to attenuate the dystrophic phenotype and enhance angiogenesis.

## Introduction

### Duchenne muscular dystrophy and therapeutic approach

Duchenne muscular dystrophy (DMD), an X-linked genetic disorder that primarily affects males, initially characterized by progressive muscle weakness, develops into generalized weakness and hypertrophy of multiple muscle groups. DMD is caused by a defective gene named *dystrophin* (*DMD*) located on the X chromosome, which is responsible for the production of dystrophin protein^1–3^. In studies from Europe and North America, the prevalence of DMD ranges from 1.3 to 2.1 per 10,000 live male births^4,5^. Although histological and laboratory findings of myopathy may be observed from birth among male children, the clinical onset of weakness usually occurs between 2 and 3 years of age, although affected boys are late walkers^6^. The weakness selectively affects the proximal muscles before the distal muscles and the lower extremities. The affected child has difficulty running and jumping and has an unusual waddling gait and lumbar lordosis. Gower's sign (when rising from the floor, the affected boys may assume an upright position with his hands supporting the body) is a common sign in children with DMD. Patients are generally wheelchair-bound by the age of 12 years, and the incidence of symptomatic cardiomyopathy increases gradually in teens. Cardiomyopathy was described in a series of 328 boys with DMD, about one-thirteenth by age 14, one-half by age 18, and all cases over age 18^7^.

**Figure 1.**
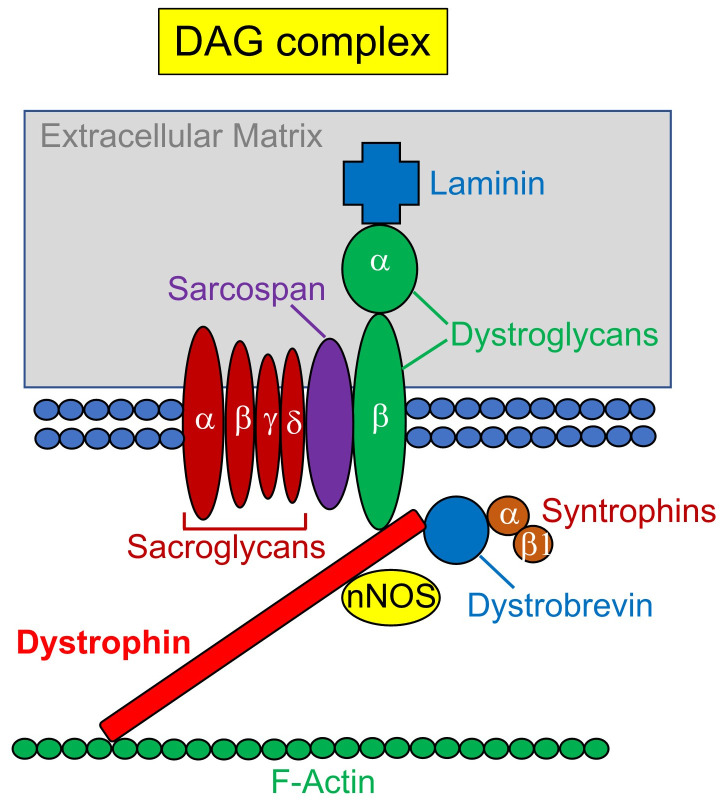
Dystrophin-glycoprotein complex (DGC). Dystrophin protein possesses a rod-shaped structure that links the intracellular cytoskeleton network to extracellular components of the dystrophin-glycoprotein complex (DGC), including dystroglycan and sarcoglycans. Aside from structural roles, the sarcoglycan subcomplex plays essential roles in signal transduction and mechanoprotection. The central mucin domain in α-dystroglycan is heavily O-glycosylated, which plays an important role in the protein function. Alteration of the dystrophin glycoprotein complex (DGC) can lead to myofiber degeneration with necrosis resulting in alteration of muscle hemostasis, including compensation of muscle regeneration and inflammation-mediated fibrosis, as the main pathophysiology in patients with Duchenne muscular dystrophy (DMD).

The *dystrophin* gene is located on the short arm of chromosome X near the p21 locus and codes for the large protein Dp427. Despite the low percentage of dystrophin protein expression (about 0.002%) of the proteins in striated muscle, its importance in maintaining the membrane integrity of muscle remains high^8^. The *dystrophin* gene, the largest gene yet identified in humans, spans approximately 2.3 megabases at chromosome Xp21.2, and the protein product has a large size (427 kDa). In about 60% of patients with DMD, *dystrophin* mutations are associated with deletions of one or more exons. The remaining cases of DMD are caused by mutations in single-nucleotide variants, small deletions, or insertion in the coding sequence, resulting in out-of-frame protein products^9–12^. These mutations lead to disruption of dystrophin protein function as a part of the dystrophin-glycoprotein complex (DGC) (Figure 1), and thus, muscle fibers become vulnerable to mechanical stretching and subsequent muscle damage. These muscle damages eventually lead to ambulation and secondary complications, including respiratory muscle and cardiac functions, and without intervention, the mean age at death is around 19 years if proper clinical treatment is not given^13^. Non-progressive cognitive dysfunction might also be present in DMD^14^.

*mdx* mice, an animal model for DMD, display continuous muscle degeneration and the following regeneration with subsequent fibrosis in the muscle. However, *mdx* mice do not exhibit the severe pathology of human DMD^15,16^. Importantly, owing to the upregulation of utrophin, a dystrophin-related protein that can compensate for the lacking function of dystrophin, *mdx* mice show no significant difference in their lifespan compared with wild-type mice. Compared with the *mdx* mice, *mdx* mice carrying a *utrophin* gene knockout (KO) (*mdx:utrn*^−/−^) display a very severe muscle phenotype and usually die within 5 months^17^.

## Vascular therapy for Duchenne muscular dystrophy

Several therapeutic approaches have been developed over the past two decades to treat DMD. Two broad categories of approaches have emerged: Targeted dystrophin-based therapies, such as gene-based, cellular-based, or protein replacement therapies, which aim to restore dystrophin protein expression and function^18^. Other therapeutic strategies include targeting downstream pathological changes, such as inflammation, fibrosis, and atrophy, via the upregulation of compensatory proteins, reducing inflammatory cascades, and enhancing muscle regeneration. Clinical trials are being conducted for several of these approaches, which are unavailable in clinical settings. However, some of these approaches have received regulatory approval in the United States, Europe, and Japan^18,19^. Most of the injured necrotic muscle fibers are observed in clusters in DMD, and the early pathophysiologic hypothesis postulated vascular changes as the culprit for the muscle injury process. Vascular dysfunction has been suggested as an underlying pathogenic mechanism of muscle injury in DMD. The deficiency of *dystrophin* in vascular smooth muscle and the absence of nitric oxide (NO) synthase from the sarcolemma has indicated that DMD muscle is prone to impaired blood flow^20^. The demonstration of decreased vascular density in the DMD model *mdx* mice and immunostaining of arterioles point toward the vascular pathogenesis in DMD^17^. Palladino *et al.*^21^ and Kodippili *et al.*^22^ showed that angiogenesis is indeed impaired in the *mdx* mouse hindlimb muscle ischemic model.

**Figure 2.**
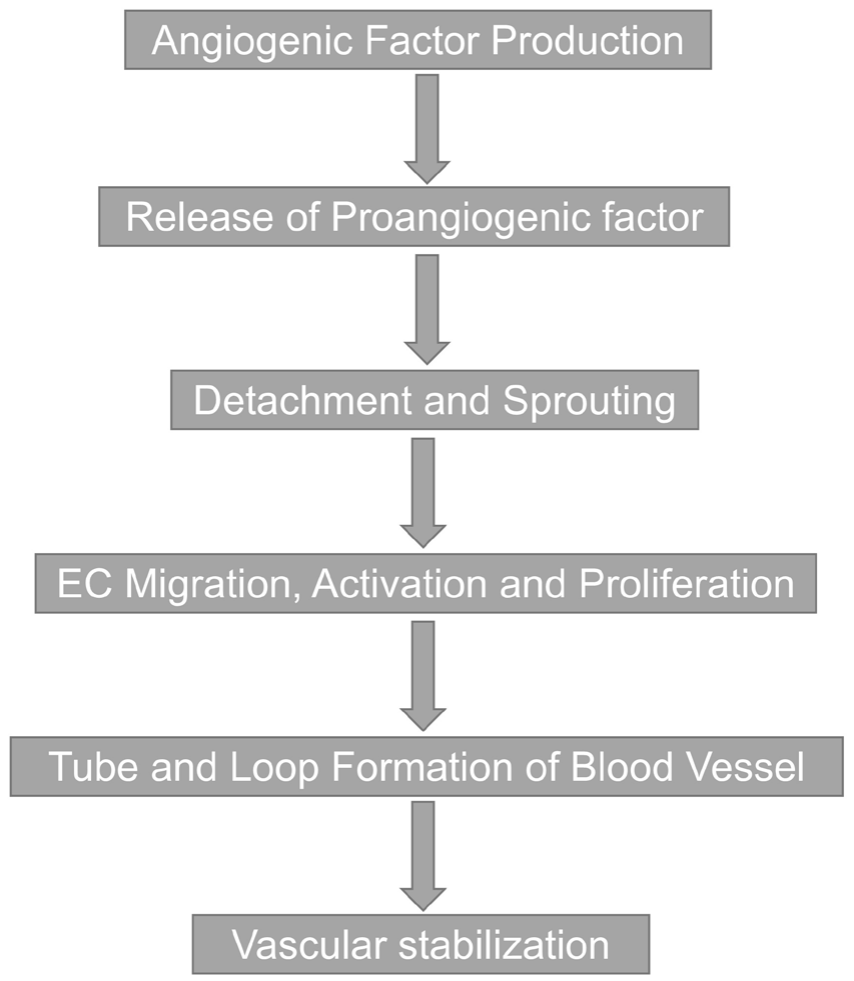
Flowchart of angiogenic formation. The steps involved in the formation of blood vessels after the production of angiogenic factors are shown. Endothelial cell (EC) migration, activation, and proliferation lead to the tube and loop formation of blood vessels, making it more stabilized.

**Figure 3.**
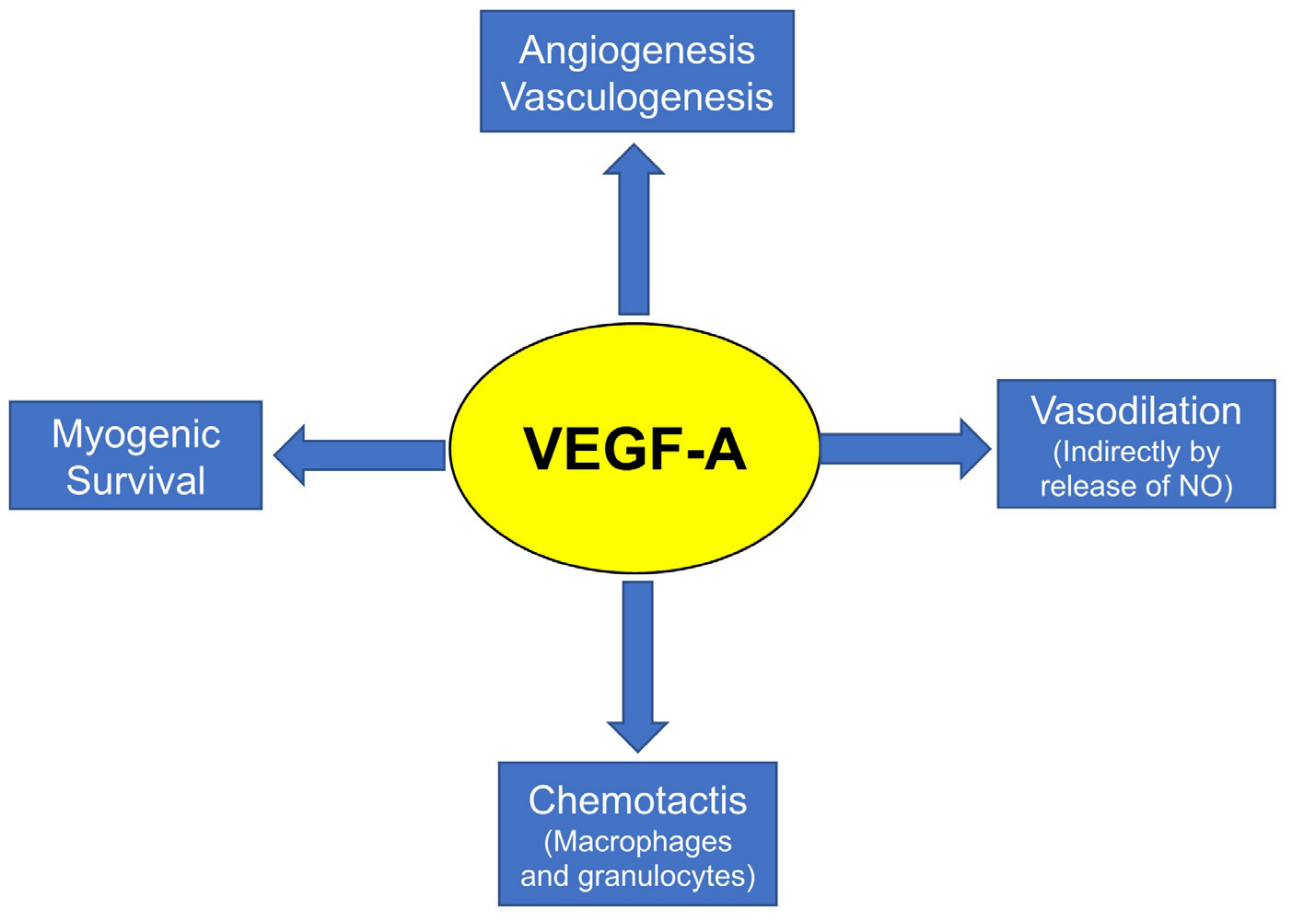
The function of VEGF-A. Vascular endothelial growth factor A (VEGF-A) is the major mediator of vasculogenesis, angiogenesis, vasodilation via nitric oxide (NO) release, chemotaxis of macrophages and granulocytes, and survival of cells, including myogenic cells. Together, these VEGF-A-mediated effects promote regeneration of skeletal muscle.

Tissue angiogenesis is a complex process that is stimulated mainly by several growth factors. Several angiogenic growth factors, such as VEGFs and fibroblast growth factors (FGFs), have the ability to bind to structurally similar glycosaminoglycans called heparin sulfate on the cell surface and in the extracellular matrix. VEGF, previously known as vascular permeability factor (VPF), is regarded as one of the most dominant growth factors for angiogenesis^23,24^. As described above, angiogenesis plays an essential role in the pathogenesis of DMD^25^. Several known pathogenetic factors, including myofiber defects and cardiomyopathy, are associated with DMD pathology and its progression, but impaired angiogenesis has been considered to be one of the major impacts of DMD pathology. Muscles, which comprise around 40% of human body mass, are solely dependent upon oxygen to function effectively^26^. There is a strong correlation between angiogenesis stimuli and VEGF^26–28^. Figure 2 demonstrates the pathogenesis or steps involved in angiogenesis after the low oxygen stimuli leading to activation of angiogenic signals, followed by vascular formation. These processes include angiogenic factor production, the release of pro-angiogenic factors, binding of angiogenic factors to their receptors, and activation of downstream signaling of the receptors, followed by detachment, and sprouting of endothelial cells (ECs), EC activation, migration and proliferation, tube and loop formation of blood vessels and vascular stabilization. Furthermore, angiogenic stimuli with VEGF-A production not only stimulate angiogenesis and vasculogenesis but also promote vasodilation, chemotaxis of macrophages and granulocytes, and muscle cell survival, followed by helping in muscle fiber regeneration (Figure 3).

**Figure 4.**
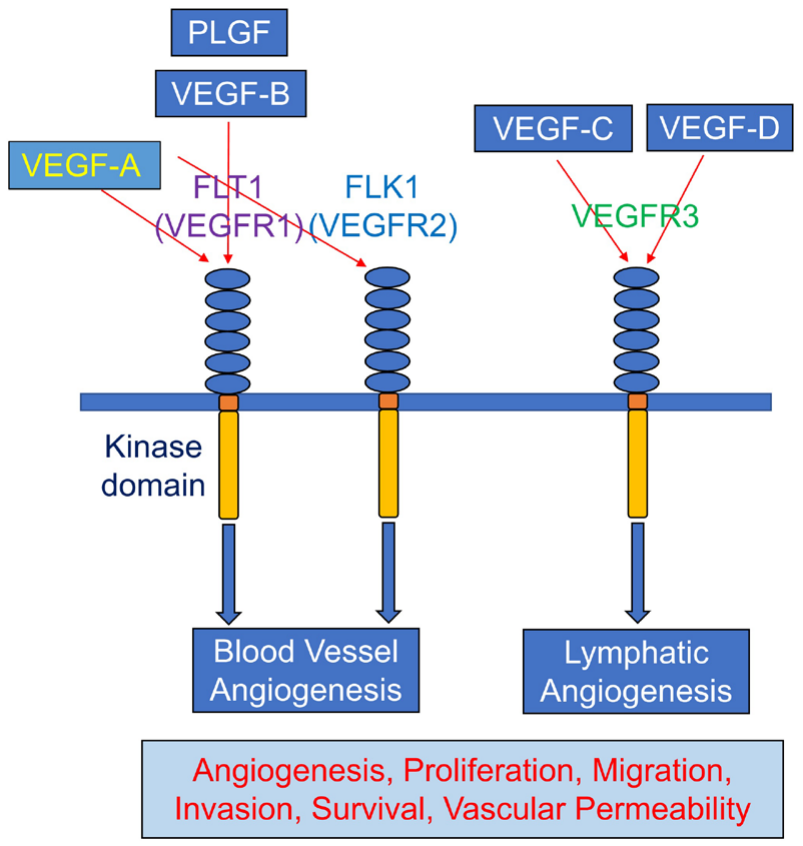
Mechanism of action of vascular endothelial growth factor. The mechanism of action of vascular endothelial growth factor, which functions through its affinity for different receptors and exerts significant effects on blood vessels and lymphatic vessels, is shown.

VEGFs are classified as VEGF-A, VEGF-B, VEFG-C, and placental growth factor (PGF or PLGF) (Figure 4). Among them, VEGF-A is regarded as the most studied and targeted pharmacological therapy. Different cell types like inflammatory and hematopoietic cells, epithelial lineages, and ECs produce VEGF-A, and it acts in vascular endothelium and helps in the process of angiogenesis *in vivo* and *in vitro*^23,24^. VEGF-A plays an essential role in the induction, growth, survival, and differentiation of vascular ECs (Figure 3). In addition, VEGF-A has essential effects on diverse biological processes, not only limited to vascular ECs but on tissue metabolism, hematopoiesis, bone formation, and pathologic hematopoiesis. Owing to the role of VEGF-A in both normal and abnormal angiogenesis, it has become an important factor for both pro-angiogenic and anti-angiogenic therapy. VEGF family acts through their receptors consisting of three different proteins, VEFGR1 (FLT1), VEGFR2 (FLK1/KDR), and VEGFR3 (FLT4), which help to mediate angiogenic signals to the vascular endothelium via binding to their extracellular domains and activating cytoplasmic tyrosine kinases (Figure 4). VEGF-A binds to this FLT1 and FLK1 and mediates different angiogenic and anti-angiogenic processes depending on the receptors. These receptors are highly expressed on ECs and characterized by seven immunoglobulin (Ig)-like domains in their extracellular margin, a single transmembrane domain, and an intracellular tyrosine kinase^27,29–31^. FLK1 plays a major role in the process of pro-angiogenesis. On the other hand, FLT1 plays a negative role in angiogenesis since VEGF has a high affinity to the FLT1 receptor but less kinase activity, making the receptor a decoy for VEGF^32,33^. Neuropilin1 (NRP1) and Neurolipin2 (NRP2) have also been described as co-receptor for FLK1, being responsible for enhancing the biological effect of VEGF on ECs.

**Figure 5.**
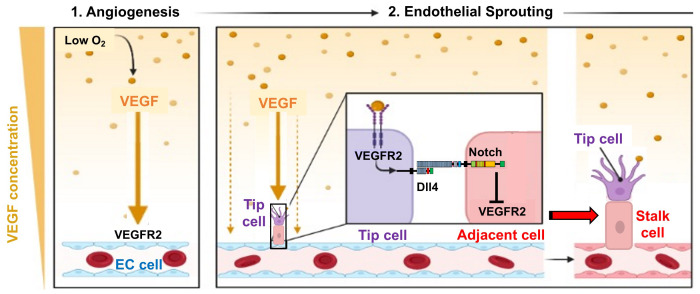
Process of sprouting angiogenesis in blood vessels. (**1**) Angiogenesis occurs in areas of low oxygenation and induces the production of vascular endothelial growth factor (VEGF), which binds to VEGFR2 on the surface of endothelial cells (ECs) to induce angiogenesis. (**2**) VEGF-VEGFR2 signaling triggers EC sprouting, which in turn causes Notch-mediated lateral inhibition in adjacent cells by the tip cells. VEGF-VEGFR2 signaling promotes tip cell differentiation while inhibiting tip cell formation in adjacent cells via the Dll4-Notch signaling pathway, promoting stalk cell differentiation of adjacent cells. This Figure was created with BioRender.com.

In adult tissues, angiogenesis occurs in areas with low oxygenation, which is a trigger to induce the VEGF gene expression and protein via transcriptional activation by hypoxia-inducible factor-1 alpha (HIF-1α) in the hypoxic tissues. Secreted VEGF binds to VEGFR2 on the surface of ECs and induces angiogenesis to form new blood vessels (Figure 5). Angiogenesis involves tip cell selection and sprout formation. The distal end of the sprout contains a tip cell, a specialized EC. VEGF-VEGFR2 signaling triggers tip cell differentiation for EC sprouting, which causes lateral inhibition of adjacent cells by the tip cells^34^. The Notch signaling pathway-mediated lateral inhibition is initially regulated by VEGF-VEGFR2 signaling, which promotes tip cell differentiation while inhibiting tip cell formation in adjacent cells via the Delta-like 4 (Dll4)-Notch signaling pathway. Tip cells express the Notch ligand Dll4, which activates the Notch pathway in adjacent cells. Notch in adjacent cells downregulates VEGFR2 gene expression, promoting stalk cell differentiation of adjacent cells and inhibiting tip cell differentiation from forming the body of the newly formed vascular vessel.

VEGF is thought to possess pro-angiogenic properties via promoting EC proliferation, survival, and differentiation. Currently, the most common method for increasing vasculature as a pro-angiogenic therapy is modulating the VEGF/VEGFR pathways^23–25^. In addition, since the targeting of VEGF-VEGFR inhibition has significant therapeutic potential for anti-cancer, recent works have used small-molecule compounds such as tyrosine kinase inhibitors that inhibit VEGFR activation and downstream signaling to explore the structural basis for anti-angiogenic therapy^35–37^. In this review, VEGF and VEGFR functions are explained in order to develop new therapeutics for DMD. In addition, we recently demonstrated that the increased vascular affects skeletal muscle regeneration as a vascular niche for muscle stem cells termed satellite cells (MuSCs) which are maintained in response to support from the vasculature as a self-renewal process^36,38^. MuSCs have been extensively characterized as a stem cell population for muscle regeneration via contribution to newly formed muscle fibers upon injury, while MuSCs possess a capacity to give rise to mesenchymal differentiation in some conditions^39,40^. The importance of considering whether therapeutically generated vasculature is beneficial from a morphological and functional standpoint cannot be overstated. Even though this issue must be thoroughly investigated before treating human DMD, increasing data indicate that angiogenesis is a promising therapeutic approach^18^.

Different studies have been carried out to show the relationship of MuSC-derived VEGF with muscle angiogenesis and regeneration. A study by Rhoads *et al.* demonstrated the relationship between VEGF and its role in muscle angiogenesis^41^. In this study, blocking VEGF in MuSC cultures decreased the capacity to promote angiogenesis *in vitro*. Verma *et al.* revealed that MuSC-derived VEGF-A is essential for proper muscle regeneration in mice^28^. More studies are to be carried out to make the relationship between vascular changes and DMD more efficient. However, a study was carried out to improve vasorelaxation capacity in the DMD model animals, leading to novel therapeutic applications^42^. Several studies have been carried out to show that the role of VEGF administration is capable of initiating angiogenic signals in skeletal muscle and both ischemic and nonischemic conditions^43^.

In this review, we reviewed the substantial advances that have been made in both lines of strategy. We focus on those that have passed the preclinical testing phase and have significant potential for clinical use. Based on preclinical and clinical research, we discuss the rationale and efficacy of each clinical approach. There are several unforeseen challenges in the current clinical trials for the treatment of DMD, including immune responses to the generated mini-*dystrophin* in the ongoing gene therapies and inconsistent evidence of dystrophin production in muscle tissues. For emerging therapies to be safe and effective in the long run, appropriate clinical endpoints and sensitive biomarkers must be selected. Many individuals with DMD and their families are finding hope in vascular-mediated therapies, including VEGF-targeted therapies. It is also necessary to target multiple disease processes in DMD since its pathogenesis is complex.

## VEGF mechanism of action

In vertebrates, VEGF and VEGFR play an essential role in regulating angiogenesis and lymphangiogenesis^44^. In the early embryonic and adult stages, VEGF activates specific receptors by binding to the extracellular domain of those receptors, which is responsible for maintaining the angiogenic balance. The de-regulation of the VEGF-VEGFR has been directly linked to several diseases, particularly cancer. Furthermore, tumor growth requires a blood supply to provide oxygen and other nutrients. A tumor’s ability to metastasize depends on blood vessels transporting tumor cells to distant sites, where they can become implanted and begin growing secondary tumors. Therefore, it is crucial to investigate signaling systems related to human diseases, such as VEGF-VEGFR, in order to develop treatments for such diseases^44,45^.

The VEGF family binds to membrane receptors on a wide range of cells to regulate various biological processes. Figure 4 shows the mechanism of action of VEGF, which exerts significant effects on blood vessels and lymphatic vessels because of its affinity for different receptors. VEGF-A, a member of the VEGF family, contributes to vascular development and function by stimulating angiogenesis. It facilitates cell migration, proliferation, survival, and tube formation^46^. VEGF-A, also one of the most potent permeability factors, serves as a common link between angiogenesis, permeability, and inflammation. During embryonic development, wound healing, and ovarian development, VEGF-A expression patterns are closely associated with the proliferation of blood vessels. Local hypoxia is a potent inducer of VEGF-A expression from ECs and adjacent cells, suggesting that vessel formation is regulated in a cell-autonomous and paracrine manner. The expression of VEGF-A is induced in fibroblasts, ECs, cardiomyocytes, skeletal muscle fibers, MuSCs, keratinocytes, macrophages, T cells, astrocytes, osteoblasts, and smooth muscle cells. In addition, VEGF-A is expressed in several human tumors^46,47^.

VEGF-A binds with high affinity to two VEGF receptor tyrosine kinases (FLT1 and FLK1) but with a lower affinity to co-receptors called NRP1 and NRP2. The FLT1 and FLK1 on the surface of the cells form homophylic dimerization and trans-phosphorylate as a tyrosin kinase upon binding to VEGF. In the endothelium of the vascular system, both FLT1 and FLK1 mediate either angiogenesis or anti-angiogenic signals^9^. The reduced kinase activity of FLT1 is thought to act as a decoy receptor for VEGF despite its high affinity for VEGF in comparison with FLK1, while the major pro-angiogenic effect is generated by FLK1^47,48^. VEGF-A/VEGFR signaling is mediated via downstream effectors to induce EC survival, proliferation, migration, maturation, and vascular permeability. Downstream effectors are p38 mitogen-activated protein kinase (p38 MAPK), phosphatidylinositol-3 kinase (PI3K), Src kinase, focal adhesion kinase (FAK), Smad2/3, and phospholipase C gamma (PLCγ)/Erk1/2^48^.

## Pro-angiogenic and pro-myogenic effects of the VEGF/VEGFR pathway in muscle

VEGF is a critical regulator of angiogenesis that is induced by hypoxia and ischemic conditions in adults. In addition to its angiogenesis-promoting properties, VEGF exhibits direct myogenic effects on skeletal muscle. It has the ability to inhibit apoptosis in myogenic cells in response to traumatic injury as well as stimulate MuSC proliferation, muscle fiber growth, and increased muscle force^49,50^. Interestingly, gene expression data reported increased FLT1 and FLK1 levels in MuSCs during induced muscle injury or local VEGF administration. As MuSCs express low steady-state levels of FLT1 and FLK1, they become substantially activated in response to VEGF stimulation, and their expression is coordinated during skeletal muscle differentiation. VEGF stimulation increases myogenic differentiation and promotes myofiber hypertrophy, whereas the addition of a soluble form of FLT1 (sFLT1), an inhibitor of VEGF, results in the inhibition of myogenic differentiation and myofiber hypotrophy^51–53^. Matrix metalloproteases (MMPs) are a family of endopeptidases involved in the degradation of extracellular matrix components, essential for embryonic development, tissue remodeling, and disease processes. Among the MMPs, MMP-2 is upregulated in skeletal muscles in DMD, and the *mmp2* gene KO in *mdx* (*mdx*:*mmp2^−/−^*) mice display impaired skeletal muscle pathology and function via reduced VEGF levels, reduced angiogenesis, and decreased myofiber growth^49^. Additional studies have shown that myofiber-specific deletion of *VEGF-A* in mice reduces capillary density, lumen dilation, and endothelium thinning of capillaries. Consequently, these KO mice display reduced exercise endurance^53–56^.

## Evidence for angiogenic alterations in animal models of Duchenne muscular dystrophy

VEGF has been implicated as a causative agent in DMD based on several studies demonstrating a role for VEGF in angiogenesis and a correlation between VEGF and vascular density. Both VEGF and HIF-1α levels in *mdx* mice were significantly lower than those in controls, suggesting that they may play a substantial role in impaired angiogenesis. However, studies that have investigated VEGF administration or vasorelaxation methods in order to improve tissue perfusion in DMD mouse models have indirectly established the role of vascular changes in the pathogenesis of DMD^57–59^. VEGF administration and VEGF receptor modulation are two treatments that aid in pro-angiogenic induction. Pro-angiogenic therapies have been shown to increase the number of MuSCs and stimulate myogenesis^19,60^. Lack of *dystrophin* in DMD may result in altered morphology and properties of the ECs, potentially altering blood vessel function^20^. Furthermore, there is a strong correlation between myogenesis and angiogenesis during skeletal muscle regeneration^61^.

DMD afflicts boys primarily; however, female carriers, with variable signs and symptoms, are also described. It has been shown that estradiol and estrogen enhance angiogenesis by increasing EC proliferation and migration. It has been suggested that this effect is mediated by the upregulation of endothelial nitric oxide synthase (eNOS) activity, increased VEGF levels, and increased expression of adhesion molecules^62,63^. There is strong evidence that sex-related differences are responsible for crucial aspects of DMD pathology, drawing attention to the possibility that female-specific factors, such as sex hormones, may play a role in these alterations^23^. As a result, sex must be carefully considered when designing experimental settings to use dystrophic mice. In the context of the disease, which affects boys, it is crucial to note that the effects seen in female *mdx* mice, also concerning the process of angiogenesis, may not necessarily be biologically relevant^64^.

## VEGF in the heart muscle and brain of murine models of Duchenne muscular dystrophy

Even though the heart is the body’s most stressed muscle, cardiomyopathy is a late manifestation of DMD. Most likely, this observation is due to differences in DGC composition and dystrophin protein expression^65,66^. It is pertinent to note that mutations that prevent the production of full-length dystrophin also prevent the production of one or shorter brain dystrophin isoforms. Consequently, cognitive impairment and neuropsychiatric symptoms are prevalent in patients with DMD. One-third of patients with DMD suffer from cognitive impairment and learning difficulties in addition to behavioral problems^67^. Dystrophin is expressed by postsynaptic inhibitory GABAergic neurons in the amygdala, hippocampus, and cerebellar Purkinje cells in the brain^67,68^. There is a fundamental role in pathological angiogenesis in the pathogenesis of neurological and cardiac manifestations of DMD. Several studies have demonstrated that *mdx* mouse brains exhibit increased levels of VEGF/FLK1 expression and increased vascularization, altering the blood-brain barrier dynamics^67–69^. Contrary to this, the downregulation of myocardial expression of VEGF in *mdx* mice resulted in decreased vascular density and quantity compared with wild-type mice^70^. Furthermore, the disturbed VEGF signaling altered coronary collateral circulation, which has a role in preventing ischemia during hypoxic conditions^71,72^.

DMD heart and brain may be protected by the modulation of angiogenesis and VEGF expression, including local cardiomyocyte transplantation. The injection of cardiomyocytes into the hearts of young *mdx*:*utrn^−/−^* mice prevented the onset of cardiomyopathy and resulted in an increase in capillaries^65,66^. In addition, another study demonstrated that mesoangioblast transplantation into the heart of *mdx* mice prevented cardiomyopathy with increased capillary density^73^. Mesoangioblasts are multipotent stem cells that possess a capacity to differentiate into the mesodermal origin, including skeletal muscle. It has been found that, compared with wild-type mice, *mdx* mice have reduced vasculature in the heart^73^. Researchers examined postnatal *Flt1* gene ablation in conjunction with left anterior descending artery ligation to create ischemic cardiomyopathy and found reduced infarction size and increased capillary density after ligation^74^. Furthermore, it was demonstrated that transplanting mesoangioblast stem cells into the heart of *mdx*:*utrn^−/−^* mice could prevent the onset of cardiomyopathy. In the treated hearts, there was an increased number of capillaries, indicating a positive effect on angiogenesis, a well-known indirect effect of stem cell therapy. The data suggest that angiogenesis may also benefit DMD cardiomyopathy^23,75^.

## A critical view on the potential use of VEGF as a treatment option for Duchenne muscular dystrophy

Previous *in vivo* and *in vitro* studies demonstrated the role of VEGF in skeletal muscle regeneration. *In vitro* studies showed that VEGF can induce MuSC migration, proliferation, survival, and myogenic differentiation via VEGFR1 or VEGFR2^51^. In addition, administration of VEGF into skeletal muscle stimulates muscle regeneration and reduces damage in ischemic muscle^76–78^. This improvement in the muscle was accompanied by increased angiogenesis and capillary density.

However, VEGF alone has not been found to be as effective as treatments in combination with other factors, such as angiopoietin-1 or insulin-like growth factor-1 (IGF-1). Therefore, an approach combining VEGF to induce angiogenesis and angiopoietin-1 (ANG-1) to mature vessels should be explored, given that VEGF alone is known to result in leaky blood vessels^79^. Moreover, enhanced IGF-1 protein expression decreased the degree of fibrosis typically observed in the diaphragms of aged *mdx* mice^80^. A reduction in myonecrosis was also found in the diaphragms and quadriceps of transgenic mice compared with age-matched *mdx* mice. Furthermore, the transgenic mice exhibited elevated signaling pathways associated with muscle regeneration and protection against apoptosis^81,82^. Intriguingly, IGF-1 inhibited the expression and activity of MIF, HMGB1, and nuclear factor kappa B (NF-κB)^83^. The US Food and Drug Administration (FDA) has approved IGF-1 to treat severe primary IGF deficiency. Phase II clinical trials of recombinant IGF-1 have been initiated in patients with glucocorticoid-treated DMD to stimulate its ability to preserve muscle function over 6 months^84^.

Another approach would be to overexpress VEGF, which can be targeted in various ways. Among these methods is delivering AVs (adeno viruses) and AAVs (adeno-associated viruses) into dystrophic mice, which has demonstrated a regenerative effect on skeletal muscle tissue, diminished necrosis, and increased capillary density^53,85,86^. Remarkably, intramuscular administration of AAV-VEGF in *mdx* mice for 4 weeks improved characteristic features of the pathophysiology, suggesting a positive outcome in the pro-regenerative effect in skeletal muscle of the DMD model mice. The muscles treated with AAV-VEGF showed enhanced gene expression and immunolocalization of its receptor, FLK1, 1 month after injection. VEGF-treated *mdx* mice showed an increased forelimb strength, which was normalized to weight in 4 weeks. The treatment reduced necrotic myofibers and increased regenerating myofibers, and increased the number of myogenin-positive MuSCs and myonuclei and myosin-heavy chain-positive myofibers^53^.

A study performed by Deasy *et al.* with VEGF overexpression via muscle-derived stem cell (MDSC) transplantation into 8 to 10 weeks of *mdx* mice showed an increase in angiogenesis with increased muscle regeneration and reduction in fibrosis^87^. In contrast, overexpression of sFLT1, a negative regulator of angiogenesis, had the opposite effects^87^.

A VEGF administration approach has been performed in combination with *dystrophin*-gene therapy for DMD, which seeks to restore the missing *dystrophin* by restoring a functional copy of this protein or repairing that protein. Viral vectors have been used to deliver a copy of the gene for a functional dystrophin protein to the affected tissues. AAVs are an exception and can infect these tissues very efficiently, even though most viruses do not have a natural affinity for skeletal muscles and the heart. The functional truncated version of *dystrophin* genes, micro- or mini-*dystrophin* constructs, which can be expressed in AAV vectors, have been generated. The expression of these micro- or mini-*dystrophin* constructs has been shown to improve pathology in mouse models of DMD^88,89^. The administration of recombinant VEGF as a VPF was used to achieve acute permeabilization of the peripheral microvasculature, resulting in enhanced tissue transduction at lower doses of micro-*dystrophin* AAV vector^90^. The combined delivery of the human mini-*dystrophin* and human VEGF genes to the muscles using AAV vectors displays synergistic improvement of muscle pathology and function in *mdx:utrn^+/−^* mice, coincident with the increased restoration of DGCs and nNOS, and reduced inflammatory cell infiltration, central nucleation, and fibrosis^86^. While the preliminary results are encouraging, AAVs rarely integrate into the host genome, and there is a possibility that the micro- or mini-*dystrophin* transgene will eventually disappear with muscle turnover. Additionally, the function of micro- or mini-*dystrophin* in humans and their corresponding therapeutic effects are under way.

Importantly, as systemic VEGF clearance is rapid, one might hypothesize that the beneficial effects of VEGF would be very short-lived and, therefore, frequent administration of VEGF would be needed to sustain VEGF-induced changes in angiogenesis and myogenesis. VEGF may need to be delivered frequently because of rapid clearance from the system. The short-term treatment with low doses of VEGF does not improve blood flow. In a study, sustained delivery of the factors through injectable gels was adequate, but bolus delivery was not associated with angiogenesis, regeneration, or muscle perfusion^91^.

Furthermore, VEGF treatment caused higher skeletal muscle collagen deposition and a pro-fibrotic response in fibroblasts isolated from *mdx:utrn^+/−^* mice. The pro-fibrotic response of skeletal muscle fibroblasts isolated from 10-week-old *mdx:utrn^+/−^* mice was observed in response to VEGF administration^82^. In conclusion, findings suggest that fibroblasts from different muscles respond differently to various growth factors. Accordingly, further research is necessary to determine whether diaphragm-derived fibroblasts respond to VEGF treatment differently than hindlimb muscles. Given that pathology progression is faster in the diaphragm than in the hindlimb muscles, future research must consider the effect of treatment on both muscles.

Currently, both VEGF- and anti-VEGF therapies have been widely examined in several different clinical trials, including trials of amyotrophic lateral sclerosis (ALS) and cancer therapy. However, the administration of VEGF may result in serious adverse effects, including vascular endothelial tumors. Furthermore, the frequency of administration of VEGF should be investigated in order to maintain desired results in contrast to side effects. As VEGF is expressed unregulated, unfavorable changes in muscle morphology may occur, indicating the necessity of regulating the transgene expression in long-term viral vector-mediated VEGF gene transfer procedures. Even though promising results have been demonstrated regarding the use of VEGF to alleviate ischemia, it has been widely claimed that while VEGF induces angiogenesis, it results only in immature and “leaky” vessels that do not confer significant benefits to the muscles^85,92^.

Inhibition of FLT1, a decoy receptor of VEGF and a negative regulator of angiogenesis, provides an alternative solution to increase VEGF-A levels, promoting VEGF-A signaling through FLK1, a receptor also involved in physiological angiogenesis. We utilized a genetic reduction of *Flt1* gene in *mdx* and *mdx:utrn^−/−^* mice. Heterozygous *Flt1* gene KO (*Flt1^+/−^*) mice were viable and displayed developmentally increased capillary density in the skeletal muscles. We have demonstrated that *mdx* mice crossed with *Flt1^+/−^* mice (*mdx:Flt1^+/−^*) improve the DMD-associated skeletal muscle phenotype as measured by increased muscle vascular density and perfusion, reduced fibrosis calcification in comparison with control *mdx* mice^93^. Furthermore, *Flt1* has also been assessed in another DMD mouse model: *mdx:utrn^−/−^* mice which display a more severe, progressive form of muscular dystrophy. Long-term studies using the *mdx:utrn^−/−^:Flt1^+/−^* mice with increased capillary density showed significant increases in body mass and survival compared with the *mdx:utrn^−/−^:Flt1^+/+^* control mice^93^. We also confirmed that postnatal EC-specific *Flt1^−/−^* mice via *Flt1-Floxed* in the *mdx* mice increased serum VEGF levels and vascular density, increased MuSC number, and improved the dystrophic phenotypes^94^.

Based on these findings, inhibiting FLT1 may benefit patients with DMD. Therefore, we performed an intravenous injection of anti-FLT1 monoclonal antibody (mAb) to *mdx* mice. Four weeks after injection, FLT1 mAbs inhibited the binding of VEGF to FLT1, increasing serum VEGF levels in the *mdx* mouse model of DMD. These mice display increased capillary density, muscle perfusion, and MuSC population and reduced fibrosis. Eventually, these treated *mdx* mice display improvement in muscle function^94^. Next, we used phage display techniques to develop a MAb (nanobody) against llama-immunized human FLT1 for therapeutic application. The lead nanobody (21B3) had a high affinity for FLT1 and efficiently inhibited VEGF binding in a competitive manner. After injection of 21B3, both the hindlimb muscle and the diaphragm exhibit significantly reduced centrally nucleated fibers, improved fiber stability, and enhanced forelimb grip strength in mice treated with 21B3 compared with control. A humanized form of the mAb, 27H6, was engineered for clinical purposes and demonstrated similar pharmacological effects^95^. In conclusion, treatment with anti-FLT1 MAbs improved muscle pathology and function in *mdx* mice. Nevertheless, for FLT1 modulation therapies, further investigation of observed toxicities, such as a vascular leak, tumorigenic progression, disorganized angiogenesis, and side effects on the other organs, should be undertaken.

## Nitric oxide-mediated Duchenne muscular dystrophy therapy

**Figure 6.**
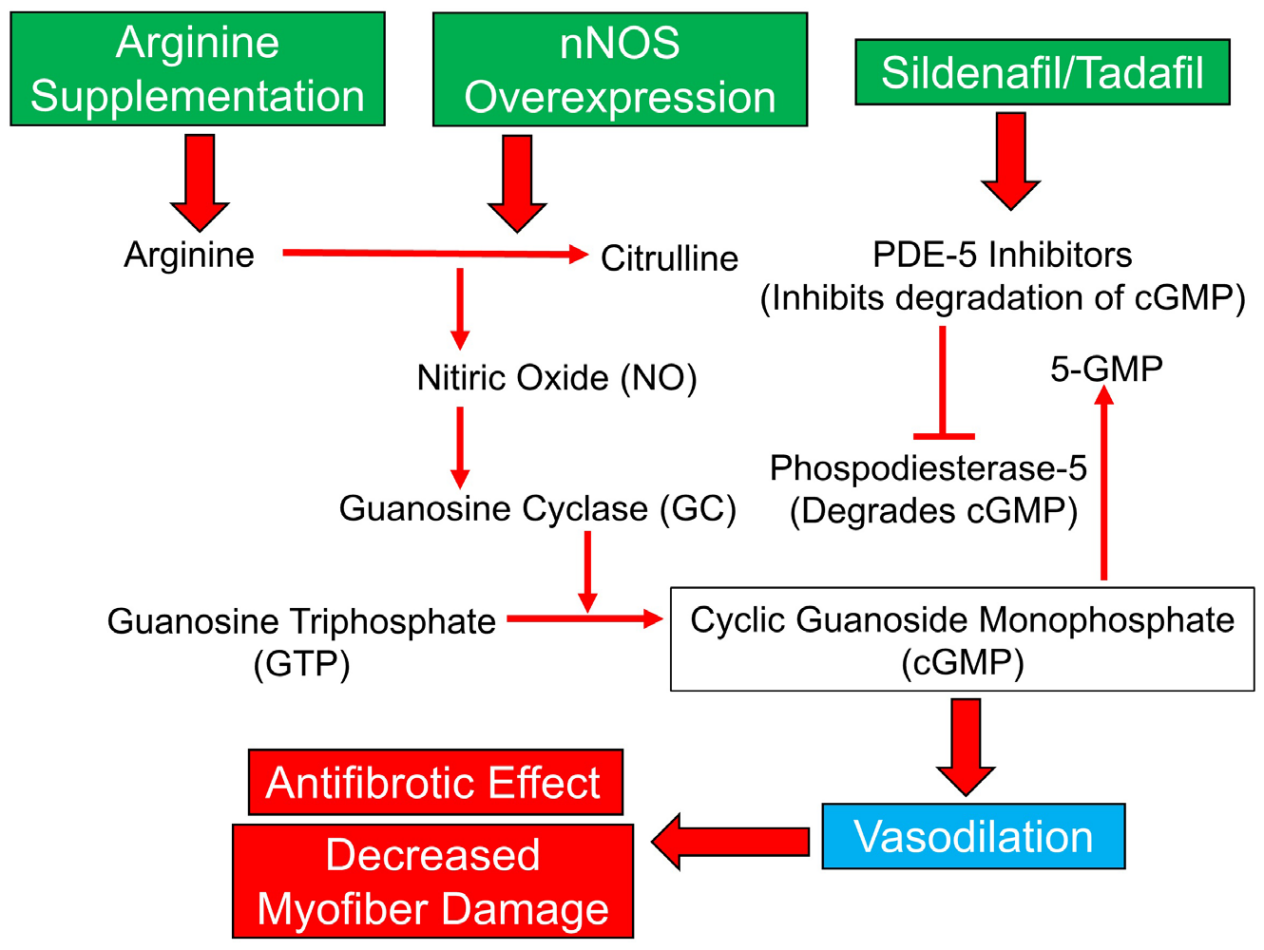
Flowchart of the production of nitric oxide (NO) for vasodilation. The steps in the production of NO leading to the vasodilation of blood vessels are shown. Meanwhile, different drugs and effect of NO are shown with their respective mechanisms of action.

Improved capacity of vasorelaxation could also be proposed as the DMD therapy. NO acts primarily by activating soluble guanylyl cyclase to increase the level of cyclic guanosine monophosphate (cGMP). Consequently, protein kinase G (PKG) is activated to induce vasodilation^96^. Thus, 5-phosphodiesterase (PDE5) inhibitors, such as tadalafil and sildenafil, which inhibit degradation of cGMP and promote production of NO, could benefit treatment of DMD and increase blood flow via vasodilation properties (Figure 6). Of note, the activity of PDE5 in *mdx* mouse muscles is age-dependent^97,98^. Young *mdx* mice had higher cyclic adenosine monophosphate (cAMP), cGMP, and PDE activity than age-matched controls. However, the older *mdx* mice (15 weeks of age) display similar cGMP PDE activity but lower cAMP PDE activity than controls^59,99^. Therefore, modulation of the NO pathway via different mechanisms, such as arginine supplementation, overexpression of neuronal nitric oxide synthase (nNOS), and PDE5 inhibitors, has been examined to show the beneficial effects on patients with DMD^100–103^. Drugs commonly acting in the below pathway of NO production help to increase the NO levels, decrease myofiber damage, and increase vasodilation effects. They also help in reducing cardiomyopathy in patients with DMD. In addition, NO pathway modulators have anti-oxidative and anti-fibrotic effects in *mdx* and *mdx:utrn^−/−^* mice^104^. Early works demonstrated that treatment with sildenafil in *mdx* mice ameliorated age-related cardiomyopathy and reduced diaphragm weakness and fibrosis^98,101^. Significantly, the tadalafil or sildenafil administered to *mdx* mice increased muscle blood perfusion and ameliorated muscle damage^105,106^. Finally, both tadalafil and sildenafil alleviated exercise-induced skeletal muscle ischemia in patients with DMD and were reported to be a putative new therapy for DMD^107,108^. Also, a PDE4 inhibitor (piclamilast) and a combination of PDE4 and PDE5 inhibitors were also found to exert beneficial anti-fibrotic effects in *mdx* mice^107^. Multiple studies suggest that PDE inhibitors are potential drugs that improve muscle pathology and performance and possibly slow disease progression in dystrophic muscles.

L-arginine, the substrate for nNOS, also possesses a role in alleviating the dystrophic muscle in DMD. Hnia *et al.* reported that *mdx* mice treated with 200 mg/kg arginine for 2 weeks displayed lower inflammation (decreased NF-κB activation and lower interleukin 1 beta [IL-1β], IL-6, and tumor necrosis factor alpha [TNFα] levels) and stabilization of utrophin/β-dystroglycan interactions as well as induction of the recruitment of nNOS in the sarcolemma^102^. In contrast, increased fibrosis in hearts and muscles was observed, so long-term (17 months) supplementation with arginine failed to protect *mdx* mice^103^. An intraperitoneal administration of arginine butyrate to newborn and adult *mdx* mice improved the dystrophic phenotype of the skeletal muscles and diaphragm^109^. In addition, oral administration of arginine butyrate improved cardiac function, decreased kyphosis, and neuronal changes in *mdx* mice^109^. In several studies, a beneficial effect of nNOS overexpression in *mdx* mice was revealed: in a study by Bloom, for example, the reduction in an inflammatory reaction with decreased macrophage and neutrophil infiltration as well as the anti-fibrotic effect in dystrophic mice was observed^96^. Improved vasorelaxation capacity via modulation of the NO signaling pathway might also be suggested as the therapy for DMD.

## Different approaches that can increase vascular density in muscle

The estrogen-related receptor-gamma (ERRγ) is an orphan nuclear receptor and is considered a master switch for oxidative and angiogenic factors. Skeletal muscles highly express *ERRγ*, which can play a significant role in muscle angiogenesis via inducing angiogenic factors like VEGF. A downregulation of *ERRγ* is observed in *mdx* mice along with its target metabolic and angiogenic genes^110^. In *mdx* mice, overexpression of *ERRγ* improved sarcolemmal integrity and muscle perfusion while restoring metabolic and angiogenic genes. Matsakasa *et al.* reported that overexpression of *ERRγ* can enhance vasculature and blood flow with an increased number of oxidative myofibres and improve exercise tolerance^111^. These data suggest that overexpression of *ERRγ* early in developing dystrophic skeletal muscles may provide a potential therapeutic strategy to overcome metabolic and pathological complications^111,112^.

In an animal study, treatment with commonly used nonsteroidal anti-inflammatory drugs (NSAIDs) that inhibit cyclooxygenase (COX) enzymes had only partial therapeutic effects. In response to arachidonic acid, COXs produce prostaglandins and lipid autacoids, contributing to pathogenic mechanisms, including inflammation. It is hypothesized that NSAIDs such as aspirin that target specific inflammatory factors may be more effective and have fewer side effects than steroidal drugs for DMD. The use of NSAIDs such as aspirin and ibuprofen on a daily basis significantly improved muscle morphology and reduced macrophage infiltration and necrosis but did not modify the percentage of regenerating myofibers in *mdx* mouse muscle. Aspirin, however, did not reduce fatigue resistance, whereas ibuprofen did. A COX-2-selective inhibitor, parecoxib, was not effective against DMD pathogenesis^113^. Aspirin is thought to have pro-angiogenic effects due to its ability to stimulate the NO cGMP pathway to enhance NO release from ECs, especially with long-term administration. Palladino *et al.* showed that the use of aspirin in *mdx* mice for 7 months showed an increase in vascular density with an increased number of regenerative fibers^21^. While COX-2 is being contrasted with muscle, Cox-2 is primarily activated in leukocytes. Additionally, steroids inhibited COX-2 in leukocytes with a more potent effect than COX-1, with only partial efficacy^114,115^.

## Statins-mediated angiogenesis for Duchenne muscular dystrophy therapy

Statins are 3-hydroxy-3-methylglutaryl coenzyme A (HMG-CoA) reductase inhibitors that are lipid-lowering agents and commonly used drugs for treating high blood low-density lipoprotein cholesterol levels, especially in cases of hypercholesterolemia and associated cardiovascular diseases^116,117^. In addition, statins have been shown to possess the potential to promote angiogenesis: low statin doses are able to promote angiogenesis through Akt activation and enhance NO production. However, multiple studies indicate that the pro-angiogenic effect of statins is dose-dependent^118^. At high dosages, an anti-angiogenic effect was observed. Thus, it is necessary to analyze the doses of statins used in various experiments. Simvastatin, the most commonly used statin, has been shown to reduce inflammation, oxidative stress, and fibrosis through a cholesterol-independent mechanism^116^. In *mdx* mice, statins have anti-inflammatory and anti-oxidative stress effects as well as reversible effects upon pre-existing fibrosis. The results of one study showed that a moderate daily dose of simvastatin significantly increased muscle strength and substantially reduced fibrosis, inflammation, and oxidative stress, three of the key pathogenic factors implicated in functional impairment and skeletal muscle damage in DMD^117^. A substantial improvement in the physiological function of skeletal muscle in terms of both force production and resistance to fatigue resulted from these mechanistic effects. These results shed light on the effectiveness of targeting specific pathogenic pathways, including oxidative stress, inflammation, and fibrosis, which are all significant contributors to the pathophysiology of the disease^117^. An additional study demonstrated no significant changes in inflammation, fibrosis, and angiogenesis, while centrally nucleated myofibers were increased after treatment with simvastatin^119^. Therefore, it is now necessary to explore the molecular and cellular mechanisms that account for these positive or negative effects. Clinically, several statins, including simvastatin, are already FDA-approved for treating familial hypercholesterolemia in children as young as 10. Simvastatin and possibly other statins may therefore be able to provide DMD and related neuromuscular diseases with readily available therapy. Owing to the immediate need for therapies that are accessible to all patients with DMD, simvastatin has significant potential to serve as a cost-effective and readily available therapy, regardless of age or specific *dystrophin* mutation^117,119,120^.

## Conclusions

A growing body of evidence has supported vascular involvement in the pathogenic process of DMD. This review concentrated on a number of new therapeutic possibilities that explicitly target these vascular abnormalities found in DMD, including functional ischemia. Treatment methods targeting vasculatures and underlying mechanisms could relieve the symptoms of DMD and even cure the disease. Modulating VEGF/VEGFR pathways is now the most popular strategy to improve vasculatures. DMD muscle may benefit from promoting angiogenesis in a variety of ways. For example, increasing capillary density improves ischemia occurring in DMD muscle. Increased vascular niches where satellite cells reside may promote muscle regeneration in DMD. While angiogenic therapies are a viable treatment approach for DMD, more research evidence is required before treating human DMD. New findings and treatment methods in DMD together may cure the disease shortly. Complementary treatment methods likely will give relief to some symptoms of DMD and delay the age of death but will not cure it. If DMD could be efficiently improved via new therapeutic approaches, it would open doors to treating other muscular dystrophies.
